# Comparison of colonic stenting and stoma creation as palliative treatment for incurable malignant colonic obstruction

**DOI:** 10.1002/jgh3.12800

**Published:** 2022-08-01

**Authors:** Sukit Pattarajierapan, Chatiyaporn Manomayangoon, Panat Tipsuwannakul, Supakij Khomvilai

**Affiliations:** ^1^ Surgical Endoscopy Colorectal Division, Department of Surgery, Faculty of Medicine Chulalongkorn University Bangkok Thailand

**Keywords:** colon cancer, intestinal obstruction, palliative therapy, self‐expandable metal stent, surgical stomas

## Abstract

**Background and Aim:**

Self‐expandable metal stent (SEMS) is a favorable therapeutic option for patients with incurable malignant colonic obstruction (MCO). However, their long‐term efficacy and safety compared with those of stoma creation have not been well investigated. This study aimed to compare these long‐term outcomes between these two techniques in patients with incurable MCO.

**Methods:**

This retrospective cohort included patients with incurable MCO with SEMS insertion (*n* = 105) and stoma creation (*n* = 97) between January 2009 and December 2019. The primary outcomes were patency after the procedure and 1‐year re‐intervention rates.

**Results:**

The patency of the SEMS group was lower than that of the stoma group (88.9 *vs* 93.2% at 6 months, 84.1 *vs* 90.5% at 12 months, and 65.8 *vs* 90.5% at 18 months; log‐rank test, *P* = 0.024), but 1‐year re‐intervention rates were not different between the groups (10 *vs* 8%, *P* = 0.558). The median patency durations were 190 days for SEMS insertion and 231 days for stoma creation. Majority (84%) of SEMS patients did not require any re‐intervention until death. The early complication rate did not differ between the groups (*P* = 0.377), but SEMS insertion had fewer late minor complications than stoma creation (5 *vs* 22%, *P* = 0.001).

**Conclusion:**

SEMS insertion is a safe and effective treatment for patients with incurable MCO. Although SEMS insertion had a lower patency than stoma creation, especially after 1 year, the 1‐year re‐intervention rates were not different, and SEMS durability was sufficient in most patients.

## Graphical abstract

Self‐expandable metal stent (SEMS) insertion is a safe and effective treatment for patients with incurable malignant colonic obstruction. Although SEMS insertion had a lower patency than stoma creation, especially after 1 year, the 1‐year re‐intervention rates were not different, and SEMS durability was sufficient in most patients.

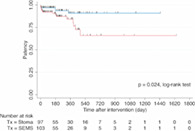

## Introduction

Colorectal cancer (CRC) is the fourth most common malignant disease worldwide, with more than 1.9 million cases in 2020.^1^ Malignant colonic obstruction (MCO) is the most frequent condition requiring aggressive management of patients with incurable CRC, being reported in 10–26% of metastatic CRCs.^2^

The three main therapeutic approaches for MCO with unresectable metastatic disease are primary tumor resection, stoma creation, and self‐expandable metal stent (SEMS) insertion. Treatment goals in the palliative setting are to relieve the obstruction, start palliative chemotherapy as quickly as possible for tumor growth stasis, and maintain quality of life.^3^ Primary tumor resection is a major abdominal surgery associated with high morbidity, prolonged recovery time, and delayed chemotherapy.^4^ Therefore, stoma creation and SEMS insertion may be preferred over primary tumor resection in patients without signs of perforation. In 2020, the European Society of Gastrointestinal Endoscopy (ESGE) guideline recommends SEMS insertion as the preferred treatment for incurable MCO based on better short‐term outcomes and quality of life than palliative surgery.^5^ However, the main drawbacks of SEMS insertion are questionable long‐term patency and possible devastating complications. A study reported an estimated SEMS patency rate as low as 50% at 12 months.^6^

Previous studies usually compared SEMS insertion with palliative surgery, including both primary tumor resection and stoma creation.^7^, ^8^, ^9^, ^10^, ^11^, ^12^, ^13^, ^14^, ^15^ Therefore, these studies could not compare outcomes between SEMS insertion and stoma creation. Relatively few studies have compared the efficacy and safety of SEMS insertion to stoma creation for incurable MCO. Moreover, the populations of these studies were small.^16^, ^17^, ^18^, ^19^ Hence, the aim of this study was to compare the long‐term efficacy and safety of SEMS insertion and stoma creation in patients with incurable MCO.

## Methods

The study protocol was approved by the Institutional Review Board (IRB) of Chulalongkorn University (IRB number: 599‐64). The requirement for consent was waived by the ethics committee because of the retrospective design of the study.

### Study design and population

We conducted a single‐center retrospective study using a prospectively maintained database of patients with incurable MCO. We included all patients who underwent SEMS insertion or stoma creation with palliative intent at King Chulalongkorn Memorial Hospital between January 2009 and December 2019. Patients who underwent primary tumor resection, intervention with curative intent, had signs of peritonitis or perforation, colonic ischemia, previous colonic stenting, or contraindication to endoscopic treatment were excluded.

MCO was defined as the presence of at least one obstructive symptom (distended abdomen, obstipation, or nausea/vomiting) and radiological findings (dilated colon proximal to the tumor) or endoscopic findings of MCO between the cecum and rectosigmoid junction. The tumor location of the enrolled patients was determined by the radiologists using an abdominal computed tomography scan. Assessment of non‐resectable liver and lung metastases was performed by a multidisciplinary team, including hepatobiliary and cardiothoracic surgeons. After discussion with the patients, the on‐call consultant colorectal surgeon decided whether to perform SEMS insertion or a surgical intervention. In addition to surgeons' preference regarding patients' condition, other important factors in decision‐making were the patient's financial status and medical reimbursement. In Thailand, SEMS insertion has been covered for reimbursement only in patients with the Civil Servant Medical Benefit Scheme. Patients with other medical benefit schemes must pay $1000 for SEMS insertion if they undergo colonic stenting.

### SEMS insertion technique

Our technique of SEMS insertion has been described in detail elsewhere.^20^ All procedures were performed in the operative theater with the patient under conscious sedation. We inserted the SEMS under fluoroscopic and endoscopic visualization. Following colonoscopic assessment of the obstructed site, a 0.035‐inch soft‐tipped hydrophilic Jagwire (Boston Scientific, Washington, DC, USA) was passed through the strictured lumen under fluoroscopic guidance. At our center, we only use uncovered colonic stents of one size, that is, 120 mm in length and 24 mm in diameter (Niti‐S D‐type, Taewoong Medical Co., Gyeonggi‐do, South Korea). We assessed the obstructed lesion preoperatively using computed tomography to ensure that a SEMS measuring 120 mm in length would be adequate. During deployment, we focused on the distal end of the stent, which was placed 30 mm distal to the tumor, and monitored the shape of the proximal end of the stent using fluoroscopy. The distance from the tumor to the distal end of the stent decreased to 20 mm after complete deployment due to SEMS foreshortening.

### Stoma creation technique

All procedures were performed with the patient under general anesthesia. The stoma sites were located within the rectus sheath to reduce the risk of parastomal herniation. For lesions distal to the splenic flexure, we preferred transverse loop colostomy over loop ileostomy because it is associated with less frequent dehydration and electrolyte imbalance. During the procedure, the previously marked site was excised as a circular skin disc approximately 2.5 cm in diameter without a midline incision if possible.

### Outcomes and definitions

The primary outcomes of this study were patency after the procedure and the need for re‐intervention within 1 year. Patency was defined as the interval from the date of intervention for obstruction relief to the date of SEMS or stoma dysfunction that required surgical or endoscopic re‐intervention. The secondary outcomes were success rates, complication rates, hospital stay, 30‐day mortality, time to initiation of chemotherapy, and overall survival (OS). In the SEMS group, technical success was defined as successful stent deployment with fluoroscopic confirmation. Clinical success was defined as the resolution of obstructive symptoms with a stool/flatus passage and oral diet tolerance. In the stoma group, technical success was defined as the technical possibility of stoma creation, and clinical success was defined similarly to SEMS placement. Major complications were defined as complications with a Clavien–Dindo classification grade IIIa or higher. Minor complications were defined as complications with a Clavien–Dindo classification grade I or II.^21^ Early complications were defined as those that occurred within 30 days of the intervention, and late complications were defined as those that occurred after 30 days.

### Statistical analysis

The distribution of the data was determined using the De Agostino–Pearson omnibus normality test. Normally distributed data are presented as means and SDs, and nonparametric data are presented as medians and interquartile ranges. Continuous variables were compared using two‐tailed Student's t‐tests or Mann–Whitney *U* tests, and categorical variables were compared using two‐tailed chi‐square tests or Fisher exact tests, as appropriate. The patency after the procedure and OS were determined using the Kaplan–Meier method and compared using log‐rank tests. The data were analyzed using Stata, version 15.1 (Stata Corp., College Station, TX, USA). Statistical significance was set at a *P*‐value <0.05.

## Results

### Study population

**Figure 1:**
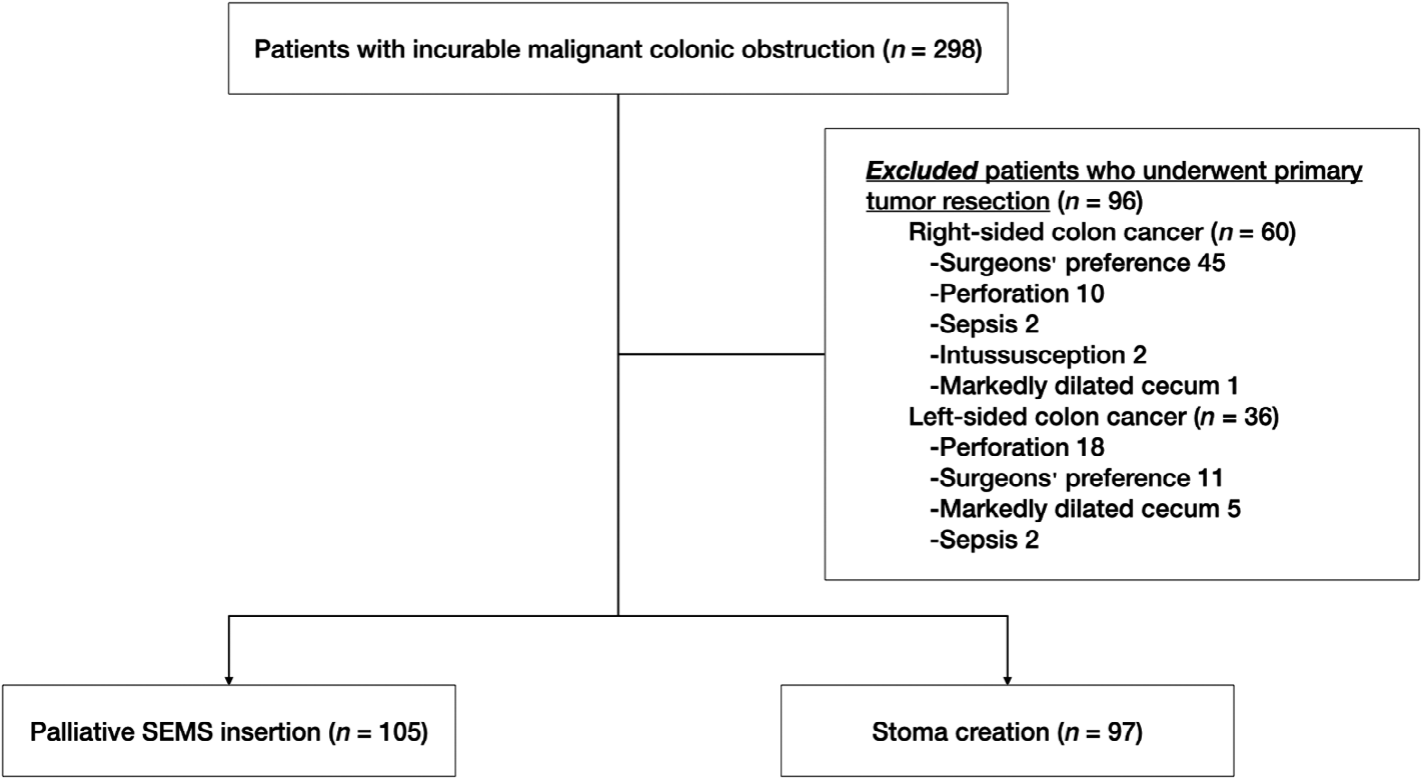
Flowchart of enrollment of patients with incurable malignant colonic obstruction. SEMS, self‐expandable metal stent.

Between January 2009 and December 2019, 298 patients with incurable MCO were identified in our database. Of these, 96 patients who underwent primary tumor resection were excluded. The reasons for primary tumor resection are shown in Figure 1. Finally, 105 patients who underwent SEMS insertion and 97 patients who underwent stoma creation fulfilled the eligibility criteria.

### Patient characteristics

Table 1 shows the baseline characteristics of the 202 patients. The patients in the SEMS group were older than those in the stoma group (mean age, 70 *vs* 63 years; *P* < 0.001). The American Society of Anesthesiologists (ASA) physical status classification of the patients in the SEMS group was higher than that in the stoma group (*P* = 0.012). There were no differences in sex, tumor location, or tumor sidedness between the groups. The patients in the SEMS group had less distant intra‐abdominal lymph node metastases than those in the stoma group (*P* = 0.024), but there were no differences in liver, lung, and peritoneal metastases between the groups. The chemotherapy access rate did not differ between the SEMS and stoma groups (53 and 58%, respectively; *P* = 0.530), but patients in the SEMS group were more likely to receive the antiangiogenic drug than those in the stoma group (15 *vs* 2%, *P* = 0.001).

**Table 1:** Baseline characteristics of the participants

	SEMS insertion (*n* = 105)	Stoma creation (*n* = 97)	*P*‐value
Age (years; mean ± SD)	70 ± 14.1	63 ± 13.9	<0.001*
Sex, *n* (%)			0.628
Male	62 (59)	54 (56)	
Female	43 (41)	43 (44)	
ASA classification, *n* (%)			0.012*
I	40 (38)	58 (60)	
II	44 (42)	30 (31)	
III	17 (16)	7 (7)	
IV	4 (4)	2 (2)	
Location of the tumor, *n* (%)			0.233
Ascending	1 (1)	1 (1)	
Transverse	8 (8)	3 (3)	
Descending	17 (16)	10 (10)	
Sigmoid	79 (75)	83 (86)	
Tumor sidedness, *n* (%)			0.256
Right	9 (9)	4 (4)	
Left	96 (91)	93 (96)	
Sites of metastases, *n* (%)			
Liver	83 (79)	83 (86)	0.226
Lung	30 (29)	30 (31)	0.714
Peritoneum	26 (25)	23 (24)	0.862
Distant intra‐abdominal lymph node	9 (9)	19 (20)	0.024*
Chemotherapy, *n* (%)	56 (53)	56 (58)	0.530
Chemotherapy regimen			
5‐Fluorouracil‐based	10 (10)	6 (11)	0.380
Oxaliplatin‐based	30 (28)	47 (48)	0.004*
Antiangiogenic drug	16 (15)	2 (2)	0.001*
Others	0 (0)	1 (1)	0.480

* *P* < 0.05.

ASA, American Society of Anesthesiologists; SEMS, self‐expandable metal stent.

In the stoma group, 87 patients (90%) underwent transverse colostomy, four patients (4%) underwent sigmoid colostomy, and six patients (6%) underwent ileostomy. We performed blowhole colostomy in two patients because of difficulty with peritoneal carcinomatosis. The remaining patients underwent loop colostomy or ileostomy.

### Primary outcomes

**Figure 2:**
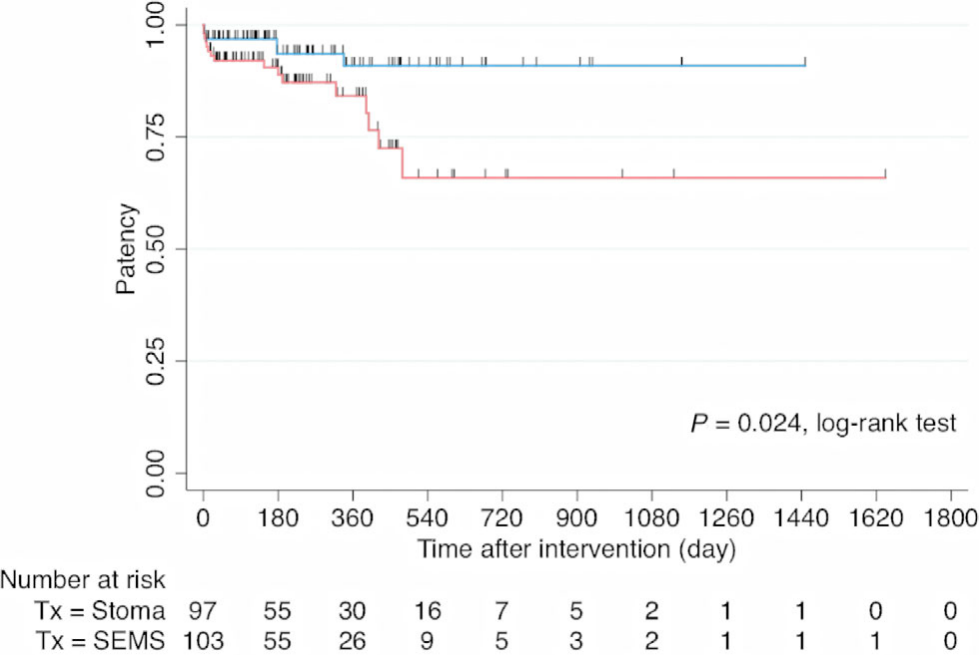
Kaplan–Meier curves of patency of patients with malignant incurable colonic obstruction who underwent self‐expandable metal stent (SEMS) insertion or stoma creation. (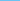), Stoma creation; (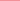), SEMS.

The patency of the SEMS group was lower than that of the stoma group (88.9 *vs* 93.2% at 6 months, 84.1 *vs* 90.5% at 12 months, and 65.8 *vs* 90.5% at 18 months; log‐rank test, *P* = 0.024; Fig. 2). The median patency durations were 190 days for SEMS insertion and 231 days for stoma creation. However, the 1‐year re‐intervention rate was not significantly different between the groups (*P* = 0.558). In the SEMS group, 84% of the patients did not require any re‐intervention until death.

### Secondary outcomes

Table 2 shows the perioperative outcomes of both groups. The technical and clinical success rates were comparable between groups. The patients who underwent SEMS insertion had fewer late minor complications than those who underwent stoma creation (5 *vs* 22%; *P* = 0.001). Meanwhile, early minor, early major, and late major complications did not differ between the groups. Details of the complications are summarized in Table 3. Seven of 105 patients (7%) had perforation after SEMS insertion. Early colonic perforation developed in five patients, and the median time to perforation was 14 days (range, 1–26 days). Late colonic perforation occurred in two patients at days 33 and 480 after SEMS insertion. Nearly all patients with perforation underwent colectomy with the Hartmann procedure, except one patient who decided to receive supportive treatment and died 3 days later. SEMS migration occurred in 8 of 105 patients (8%), and all migrations were late complications. Three patients with clinical obstruction successfully underwent SEMS reinsertion, while five patients without clinical obstruction were found with tumor shrinkage and had successful conservative treatment until their death.

**Table 2:** Perioperative outcomes of palliative self‐expandable metal stent (SEMS) insertion and stoma creation

	SEMS insertion (*n* = 105)	Stoma creation (*n* = 97)	*P*‐value
Technical success, *n* (%)	103 (98)	97 (100)	0.498
Clinical success, *n* (%)	100 (95)	96 (99)	0.214
Complication, *n* (%)			
Early			
All	9 (9)	12 (12)	0.377
Minor	1 (1)	5 (5)	0.107
Major	8 (8)	7 (7)	0.913
Late			
All	12 (11)	26 (27)	0.005*
Minor	5 (5)	21 (22)	0.001*
Major	7 (7)	5 (5)	0.770
30‐day mortality, *n* (%)	14 (13)	5 (5)	0.089
Hospital stay (days; median [interquartile range])	3 [2–7]	7 [5–12]	<0.001*
Time to chemotherapy (days; median [interquartile range])	22 [14–40]	31 [20–71]	0.011*
Re‐intervention within 1 year, *n* (%)	11 (10)	8 (8)	0.558
Eventual stoma formation rate	12 (11)	97 (100)	<0.001*

* *P* < 0.05.

**Table 3:** Complications of palliative self‐expandable metal stent (SEMS) insertion and stoma creation

Complication of SEMS insertion	Treatment
Perforation, 7 (7%)	Colectomy with the Hartmann procedure, 6 Supportive treatment, 1
Migration, 8 (8%)	Conservative treatment without clinical obstruction, 5 SEMS reinsertion, 3
Decompression failure, 2 (2%)	Colectomy with the Hartmann procedure, 1 Total colectomy, 1
Re‐obstruction, 3 (3%)	Stoma creation, 2 SEMS reinsertion, 1

Complication of stoma creation	Treatment
Stoma prolapse, 22 (23%)	Conservative treatment, 16 Incarcerated ostomy needed reduction by colorectal surgeons, 4 Revision colostomy, 2
Stoma necrosis, 1 (1%)	Revision colostomy, 1
Wound infection, 1 (1%)	Abscess drainage, 1
Bleeding, 2 (2%)	Suturing to stop bleeding, 1 Cauterization with silver nitrate, 1
Peristomal skin irritation, 2 (2%)	Peristomal skin care, 2
Parastomal hernia, 2 (2%)	Conservative treatment, 2
Partial small bowel obstruction, 1 (1%)	Conservative treatment, 1
Sepsis, 5 (5%)	Antibiotic depending on the bacterial sources, 5
Intracerebral hemorrhage, 1 (1%)	Conservative treatment, 1
Acute urinary retention, 1 (1%)	Foley catheter placement, 1

Data are presented as *n* (%).

The most common complication in the stoma group was stoma prolapse, which occurred in 22 of 97 patients (23%). Only two patients had severe prolapse that required stoma revision. The rest of the patients received conservative treatment with advice from ostomy nurses.

The median length of hospital stay was shorter in the SEMS group than in the stoma group (3 days *vs* 7 days, *P* < 0.001). The 30‐day mortality rate tended to be higher in the SEMS group than in the stoma group (13 *vs* 5%, *P* = 0.089). The time to initiation of chemotherapy was shorter in the SEMS group than in the stoma group (median, 22 days *vs* 31 days, *P* = 0.011). The eventual stoma rate of SEMS insertion was lower than that of stoma creation (11 *vs* 100%, *P* < 0.001).

**Figure 3:**
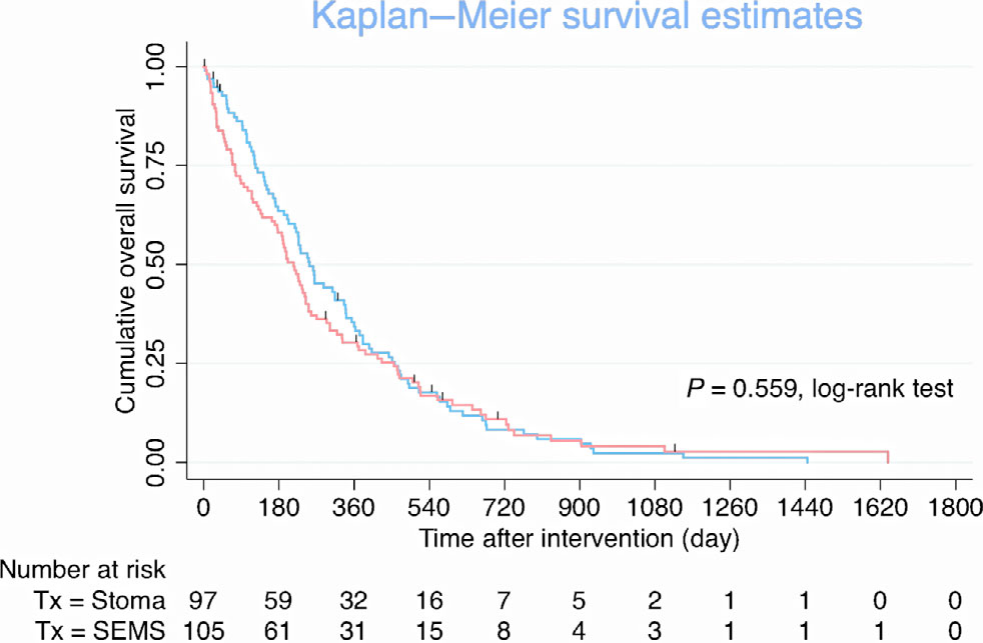
Kaplan–Meier curves of overall survival of patients with malignant incurable colonic obstruction who underwent self‐expandable metal stent (SEMS) insertion or stoma creation. (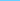), Stoma creation; (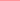), SEMS.

The median OS of all patients was 227 days. OS in the SEMS group was comparable to that in the stoma group (median, 201 and 251 days, respectively; log‐rank test, *P* = 0.559; Fig. 3).

## Discussion

This study demonstrated that palliative SEMS insertion has lower patency than stoma creation. However, the 1‐year re‐intervention rates did not differ between SEMS insertion and stoma creation. Regarding perioperative outcomes, SEMS insertion had a comparable early complication, shorter length of hospital stay, and shorter time to initiate chemotherapy than stoma creation. In the long‐term follow‐up, SEMS insertion had fewer late minor complications than stoma creation, and the most common late complication of stoma creation was stoma prolapse.

The treatment goals in patients with incurable MCO are to provide relief of the obstruction through effective and minimal morbidity, so the patient can quickly receive chemotherapy and return to their baseline quality of life.^3^ SEMS placement serves most of these goals because it is a minimally invasive procedure that maintains the ability to defecate naturally.^22^ Although the long‐term patency is questionable, SEMS patency was reported with a median duration of 106–163 days.^13^, ^17^, ^23^ Previous studies have shown that the patency of SEMS placement was shorter than that of palliative surgery.^13^, ^23^ However, no study has directly compared patency between SEMS insertion and stoma to date. In this study, although we found the patency of SEMS was shorter than stoma creation, we noticed that the patency of SEMS was not much different from stoma within the first year (88.9 *vs* 93.2% in 6 months and 84.1 *vs* 90.5% in 12 months) and 1‐year re‐intervention rates did not differ between SEMS insertion and stoma creation. Despite lower SEMS patency after 1 year, 84% of the patients who underwent SEMS placement did not require any re‐intervention until death. The explanation for this finding might be the short OS of our patients with incurable metastatic disease. In summary, SEMS is an effective therapeutic option for incurable MCO because its durability is sufficient in most patients.

Complications are a matter of concern when performing palliative SEMS insertion. These include perforation, migration, and re‐obstruction. Early complications of SEMS were observed as high as 36.5% in a meta‐analysis.^24^ Although previous studies found no difference in the complication risk between SEMS insertion and stoma creation, the populations of these studies were small.^16^, ^17^, ^18^, ^19^ Our study categorized complications into “major” or “minor” and “early” or “late” to better view the risk of these procedures. We found that SEMS insertion had fewer late minor complications than stoma creation, while the incidences of early minor, early major, and late major complications did not differ between the groups. Most of the late complications of stoma creation were stoma prolapse, which could be treated conservatively. For palliative SEMS placement, van Hooft *et al*. reported the highest early and late perforation rates of 18 and 36%, respectively.^15^ In contrast, other studies reported incidences of 0–11%.^13^, ^15^, ^23^, ^25^, ^26^ Similarly, we had a 7% incidence of SEMS‐related perforation, and most of the patients underwent emergent colectomy except one patient who chose supportive treatment and died. In our study, the antiangiogenic drug was used more in the SEMS group (15%) than the stoma group (2%), although antiangiogenic therapy has been speculated to induce SEMS‐related perforation.^27^, ^28^ The reason is Thai medical reimbursement that the antiangiogenic agent is covered in patients with the Civil Servant Medical Benefit Scheme as the second‐line treatment for incurable CRC. Therefore, patients with other medical benefit schemes were unlikely to receive antiangiogenic agents, which is similar to SEMS reimbursement. We found that 2 of 16 patients developed SEMS‐related perforation after a few weeks of initiating antiangiogenic drugs. However, examining the association between bevacizumab and SEMS‐related perforation with these limited perforation cases might not be possible. Many large studies have already shown a higher perforation rate with antiangiogenic drug, and the 2020 ESGE guideline does not suggest SEMS insertion while patients are receiving antiangiogenic therapy.^5^, ^27^, ^28^

We also found that SEMS migrations mostly occurred late and were caused by tumor shrinkage after chemotherapy, which required no intervention. On the other hand, if obstructive symptoms developed after migration, SEMS reinsertion could be performed successfully in all patients. Although previous studies showed that late SEMS dysfunction could be as high as 19%, we had only a 3% incidence of re‐obstruction from tumor ingrowth with an uncovered SEMS.^24^ In summary, the risk of major complications did not differ between SEMS insertion and stoma creation; however, SEMS insertion had fewer late minor complications than stoma creation. Prolapse was the most common late problem after stoma creation, which disturbed patients' quality of life.

Previous meta‐analyses found comparable 30‐day mortality rates between SEMS insertion and palliative surgery.^24^, ^29^ The reported 30‐day mortality rates were 6.3% in the SEMS group and 6.4% in the palliative surgery group.^24^ However, we found that patients who underwent SEMS insertion tended to have higher 30‐day mortality than those who underwent stoma creation. The reason for this might be selection bias due to the retrospective study design. The patients in the SEMS group were significantly older and had a higher ASA classification than those in the stoma creation group. Therefore, the mortality rate in this study was difficult to verify because of these confounders.

Previous systematic reviews and meta‐analyses revealed that palliative SEMS insertion was associated with shorter hospital stays and time to initiation of chemotherapy than palliative surgery, including primary tumor resection and stoma creation.^24^, ^26^, ^29^ In our study, we compared SEMS insertion with stoma creation, which has less morbidity than primary tumor resection. We found that SEMS insertion had shorter hospitalization and time to initiate chemotherapy than stoma creation. SEMS insertion is minimally invasive, and oral intake can be initiated as soon as the obstruction is resolved. Although stoma creation is a procedure with minimal major morbidity, it must be performed with the patient under general anesthesia, and oral intake is started after the sign of stoma functioning. Moreover, the patients must learn how to care for the stoma before discharge, so hospitalization is prolonged. Therefore, palliative SEMS placement is preferred over stoma creation because it shortens the hospital stay and time to initiate chemotherapy with the ability to defecate naturally, which improves patients' quality of life.

Our study has several limitations. First, since this was a retrospective cohort study, selection bias due to the consultant colorectal surgeons occurred when choosing therapeutic modalities. Older adults with comorbidities were more likely to undergo SEMS placement than younger adults. These factors might have caused a trend of worsening 30‐day mortality in the SEMS group. Second, even though we included all patients with MCO who underwent SEMS insertion or stoma creation, most of the patients in this study had left‐sided MCO. The reason for this is surgeons' preference for primary tumor resection for right‐sided MCO. Acute obstruction of the right‐sided colon can be managed by tumor resection and primary anastomosis with minimal morbidity and mortality.^5^, ^30^ Third, data on quality of life are lacking. Quality‐of‐life analysis is crucial for comparing the efficacy of SEMS placement with stoma creation. However, a previous randomized controlled trial clearly showed the quality‐of‐life benefits of SEMS placement.^22^ Fourth, there were 11 years for this study period. With this long interval, the chemotherapy and other treatment may have changed over time. Finally, it might not be possible to examine the association between bevacizumab and SEMS‐related perforation. Although two patients in our study developed perforation after SEMS placement a few weeks after the initiation of bevacizumab, the overall number of patients with perforation was limited. Larger collaborative studies may be more suitable for investigating the risk of bevacizumab.

In conclusion, SEMS insertion is a safe and effective treatment for patients with incurable MCO. Although SEMS insertion had lower patency than stoma creation, especially after 1 year, the 1‐year re‐intervention rates were not different, and SEMS durability was sufficient in most patients. Moreover, stoma creation had a high incidence of minor late complications, and stoma prolapse was the most common problem that affected patients' quality of life. Our findings indicate that for patients who are not indicated for primary tumor resection, SEMS insertion offers significant benefits compared with stoma creation.
